# Ephedra sinica polysaccharide regulate the anti-inflammatory immunity of intestinal microecology and bacterial metabolites in rheumatoid arthritis

**DOI:** 10.3389/fphar.2024.1414675

**Published:** 2024-05-23

**Authors:** Yanmiao Ma, Xiuhong Wei, Jiehao Peng, Fuxia Wei, Ya Wen, Mingran Liu, Bo Song, Yonghui Wang, Yumin Zhang, Tao Peng

**Affiliations:** ^1^ Department of Basic Medical Sciences, Shanxi University of Chinese Medicine, Taiyuan, China; ^2^ Department of Third Clinical Medicine, Shanxi University of Chinese Medicine, Taiyuan, China; ^3^ Department of First Clinical Medicine, Shanxi University of Chinese Medicine, Taiyuan, China; ^4^ Famous Chinese Medicine Studio, Shanxi Hospital of Integrated Traditional Chinese and Western Medicine, Taiyuan, China; ^5^ Shanxi Provincial Key Laboratory of Classical Prescription Strengthening Yang, Shanxi Hospital of Integrated Traditional Chinese and Western Medicine Taiyuan, Taiyuan, China

**Keywords:** rheumatoid arthritis, gut microbiota, systemic metabolism, TLR4/HDAC/NF-κB pathway, Ephedra sinica polysaccharide

## Abstract

**Introduction:**

*Ephedra sinica* polysaccharide (ESP) exerts substantial therapeutic effects on rheumatoid arthritis (RA). However, the mechanism through which ESP intervenes in RA remains unclear. A close correlation has been observed between enzymes and derivatives in the gut microbiota and the inflammatory immune response in RA.

**Methods:**

A type II collagen-induced arthritis (CIA) mice model was treated with Ephedra sinica polysaccharide. The therapeutic effect of ESP on collagen-induced arthritis mice was evaluated. The anti-inflammatory and cartilage-protective effects of ESP were also evaluated. Additionally, metagenomic sequencing was performed to identify changes in carbohydrate-active enzymes and resistance genes in the gut microbiota of the ESP-treated CIA mice. Liquid chromatography-mass spectrometry and gas chromatography-mass spectrometry were performed to observe the levels of serum metabolites and short-chain fatty acids in the gut. Spearman’s correlational analysis revealed a correlation among the gut microbiota, antibiotic-resistance genes, and microbiota-derived metabolites.

**Results:**

ESP treatment significantly reduced inflammation levels and cartilage damage in the CIA mice. It also decreased the levels of pro-inflammatory cytokines interleukin (IL)-6, and IL-1-β and protected the intestinal mucosal epithelial barrier, inhibiting inflammatory cell infiltration and mucosal damage. Here, ESP reduced the TLR4, MyD88, and TRAF6 levels in the synovium, inhibited the p65 expression and pp65 phosphorylation in the NF-κB signaling pathway, and blocked histone deacetylase (HDAC1 and HDAC2) signals. ESP influenced the gut microbiota structure, microbial carbohydrate-active enzymes, and microbial resistance related to resistance genes. ESP increased the serum levels of L-tyrosine, sn-glycero-3-phosphocholine, octadecanoic acid, N-oleoyl taurine, and decreased N-palmitoyl taurine in the CIA mice.

**Conclusion:**

ESP exhibited an inhibitory effect on RA. Its action mechanism may be related to the ability of ESP to effectively reduce pro-inflammatory cytokines levels, protect the intestinal barrier, and regulate the interaction between mucosal immune systems and abnormal local microbiota. Accordingly, immune homeostasis was maintained and the inhibition of fibroblast-like synoviocyte (FLS) proliferation through the HDAC/TLR4/NF-κB pathway was mediated, thereby contributing to its anti-inflammatory and immune-modulating effects.

## 1 Introduction

Rheumatoid arthritis (RA) is among the most common immune-mediated inflammatory diseases and is characterized by symmetrical joint pain, swelling, and stiffness. Chronic inflammation of the synovial membrane (synovium) can cause severe joint damage, disability, and work loss (Brown et al., 2024). Globally, approximately 1% of adults have RA. The exact cause of RA remains unclear. The intestine is composed of the body’s largest population of innate and adaptive immune cells and is the largest immune organ in humans. Because of the interaction between the mucosal immune system and abnormal local microbiota, the disease starts at the mucosal site and affects synovial joints (Zaiss et al., 2021).

*Ephedrae* herba is the dried herbaceous stem of *Ephedra sinica* Stapf, *Ephedra intermedia* Schrenk et C. A. Mey., or *Ephedra equisetina* Bge. It has long been used as an herbal medicine in China relieving colds, edema, nasal congestion, and rheumatoid arthritis. *Ephedra* has anti-inflammatory properties (Cao et al., 2022). *Ephedra sinica* polysaccharide (ESP) exhibits a good therapeutic effect on RA (Zheng et al., 2023) by reducing the lipopolysaccharide stimulated nuclear translocation of the NF-κB p65 subunit, improving the levels of RA inflammatory markers, and reducing the release of inflammatory factors (TNF-α, IL-1β, and IL-6) and NO by inhibiting the TLR4 signaling pathway (Li et al., 2023). Arabinose and glucose contents in plant polysaccharides directly influence their immune activities (Wang et al., 2016).

The gut microbiota is a chief determinant of immune therapeutic responses. Bacterial metabolites are associated with human physiology and disease, thereby influencing the immune function. Dietary fibers undergo fermentation by the gut microbiota, resulting in the production of short-chain fatty acids (SCFAs), including acetic acid, propionic acid, and butyric acid. These SCFAs regulate the phenotype and/or function of macrophages, neutrophils, dendritic cells, and CD4^+^ T cells (Wang et al., 2023a).

The gut microbiota can exert profound effects on the host’s metabolism. The relationship between the gut microbiota, metabolites, and clinical features in the preclinical stage of RA remains unclear (Reinke et al., 2017). Toll-like receptors (TLRs) in intestinal epithelial cells can activate kinases, including interleukin-1 receptor-associated kinase and mitogen-activated protein kinase, thereby promoting the nuclear translocation of nuclear transcription factor-κB (NF-κB), stimulating the expression of inflammatory cytokines, and increasing the permeability of the small intestinal epithelial cell layer (Luo et al., 2023). Therefore, the impact of ESP on the gut microbiota must be better comprehended, along with a clear observation of ESP metabolites and an assessment of the downstream effects exerted by metabolites on RA.

The CIA mice model was used to simulate joint damage in RA and treated the mice with ESP for 21 days. At the end of the experiment, rectal feces, serum, and synovial tissue were collected from the mice to scrutinize the gut microbiota profile, joint damage, serum metabolites, and intestinal mucosal barrier of the CIA mice. We also investigated how the gut microbiota absorbs and uses macromolecular polysaccharides and how changes occur in its microbial-derived products. The goal explore the relationship between the gut microbiota, joint inflammation damage, and host immune dysregulation in RA pathogenesis.

## 2 Materials and methods

### 2.1 Reagents

Chromatographic column (ACQUITY BEH C18100 mm × 2.1 mm, 1.7 um); mass spectrometry-grade methanol (67-56-1, Thermo Fisher Scientific); mass spectrometry-grade acetonitrile (75-05-8, Thermo Fisher Scientific); bovine type II collagen (220195, SIGMA-ALDRICH); Freund’s Complete Adjuvant (SLCL9648, SIGMA-ALDRICH); toll-like receptor 4 (A5258, ABclonal); NF-κB p65 (A2547, ABclonal); myeloid differentiation primary response 88 (67969-1-Ig, Proteintech); mouse IL-1β ELISA kit [JL18442, Sangon Biotech (Shanghai)]; mouse IL-6 ELISA kit [JL20268, Sangon Biotech (Shanghai); methotrexate [036210103, Sangon Biotech (Shanghai)]; PDTC (5108-96-3, MedChemExpress); TSA (58,880-19-6, MedChemExpress).

### 2.2 Animals

C57BL/6J mice were provided by Beijing Weitong Lihua Experimental Animal Technology Co., Ltd. (Beijing, China). The temperature was maintained at 15°C–25°C, relative humidity of 45%–55%, and a simulated natural light/dark cycle of 12 h each. All animals had free access to water and food.

### 2.3 Preparation and quality control of ESP

*Ephedra sinica* Stapf herba (401003136P) was decocted, and the filtrate obtained was concentrated to 0.5–1 g/mL. Ethanol (95%, anhydrous) was added at a 1:9 ratio to precipitate the extract for 30 min. The mixture was centrifuged at 4°C and 3,500 rpm for 10 min. This process was repeated 2–3 times. The resulting extract was filtered and subjected to rotary evaporation to obtain the ESP powder.

High-performance liquid chromatography (HPLC) was performed for the quality control of ESP. Experimental conditions for HPLC are detailed in the Supplementary Material S1.

### 2.4 Animals and treatments

After 1 week of acclimatization feeding, the mice were randomized into five groups: the natural control group (NC), collagen-induced arthritis group (CIA), methotrexate group (MTX), low ESP group (200 mg/kg), and high ESP group (400 mg/kg). Then, 1 mg/mL Type II collagen (220,195, Sigma-Aldrich) and Freund’s complete adjuvant (SLCL9648, Sigma-Aldrich) were emulsified thoroughly on an ice bath at 1:1. Then, 0.1 mL of the emulsion was injected into the tail, back, and left hind paws of the mice, whereas 0.1 mL saline was injected at the same sites in mice from the blank group. After 1 week, immunization was reinforced by injecting 0.1 mL of the emulsion at the same sites.

Saline was orally administered to the NC and CIA groups daily. The MTX group received 9 mg/kg methotrexate tablets, and the ESP-L and ESP-H groups received the ESP suspension orally for 21 consecutive days.

### 2.5 Assessment of joint swelling and body weight changes in mice

To record the changes in the left hind paw volume and body weight of the mice, weekly measurements were conducted. The volume of the left hind paw joint was precisely measured using a specialized paw volume measurement device; the average value was obtained from three measurements.

### 2.6 MTT assay for cell proliferation

The mice fibroblast-like synoviocyte (FLS) cell line (CTCC-S004-MIC, Meisen) was cultured in DMEM medium containing 10% fetal bovine serum, 1% penicillin, and streptomycin at 37°C with 5% CO_2_. FLS were seeded into 96-well plates at 8 × 10^3^ cells/100 μL/well. The medium was changed after 24 h and different ESP concentrations were added to each well for continued culture for 24 or 48 h. Then, 10 μL of the MTT solution (M8180, Solarbio) was added to each well and incubated for 4 h in a CO_2_ incubator. After 150 μL of DMSO was added to each well and crystals were allowed to completely dissolve, the optical density of each well was measured at 570 nm.

### 2.7 Scratch assay for the wound healing rate

The cells were vertically scratched using a 200 μL pipette tip and gently washed with PBS to remove detached cells. The medium was replaced with a 2% FBS-containing medium. The scratch width at 0 and 12 h was photographed under an inverted microscope (Nikon, Japan). The cell healing rate at 12 h was measured using ImageJ software.

### 2.8 Masson, Safranin O-fast green (S&F), and TRAP staining

Each joint tissue in every group was fixed with 4% paraformaldehyde, dehydrated with ethanol gradient, cleared with xylene, and 4μm sections. These slices were stained with Masson trichrome (G1340, Solarbio) to evaluate histopathological changes and collagen deposition. S&F staining (G1371, Solarbio) and TRAP Staining (G1492, Solarbio) were performed using a commercial kit. The modified Osteoarthritis International Study (OARSI) scoring system was used to evaluate the histological features of cartilage degeneration. The modified Mankin’s scoring system is used for assessing the grades of the structure and tidemark intactness.

### 2.9 Enzyme-linked immunosorbent assay

Serum levels of IL-1β and IL-6 were quantitatively analyzed using the mice ELISA kit. Absorbance was read at 450 nm by using an ELISA reader. After a standard curve was constructed, the concentration of substances in the samples was calculated.

### 2.10 Cell culture and treatment

PDTC (1 mg, 5108-96-3, MedChemExpress) was dissolved in 6.09 mL of sterile water to prepare a 1 mM reserve solution. TSA (1 mg, 58,880-19-6, MedChemExpress) was dissolved in 3.3 mL of DMSO and stored as a 1mM reserve solution.

The cells were divided into five groups, namely, the Control group, Control + PDTC group, Control + PDTC group, ESP group, ESP group, ESP + PDTC group, and ESP + PDTC + TSA group. The cells were pretreated with 100 nM IL-1β and 100 nM TNF-α for 24 h.

### 2.11 Protein immunoblotting experiment

After the tissues or cells were collected, the proteinase inhibitor (PR20032, Proteintech) and phosphatase inhibitor cocktail (PR20015, Proteintech) were added to the RIPA lysis buffer (AR0102, Bosterbio). The protein concentration was quantified using the BCA assay kit (AR0146, Bosterbio). The proteins were separated through electrophoresis on denaturing SDS-PAGE gels (AR0138, Bosterbio). The proteins were transferred onto PVDF membranes (0000215179, Millipore), blocked with 5% non-fat milk in TBST, and incubated overnight with the following primary antibodies at 4°C: HDAC1 (1:10,000, Proteintech), HDAC2 (1:10,000, Proteintech), TLR4 (1:1,000, ABclonal), MyD88 (1:1,000, Proteintech), TRAF6 (1:2000, ABclonal), NF-κB p65 (1:4,000, Proteintech), pNF-κB p65 (1:1,000, Cell Signaling), ZO-1 (1:5000, Proteintech), and β-actin (1:5000, Bioworld Technology). The membranes were washed the next day, incubated with goat anti-rabbit IgG secondary antibody (1:5000, Proteintech) for 2 h, and then visualized using the Meilunbio^®^ FG Super Sensitive ECL Luminescence Reagent (MA 0186, Meilunbio). ImageJ software was used for densitometry analysis.

### 2.12 Metagenomic analysis

DNA was extracted from the fecal samples, and its quality and purity were assessed. After the DNA was fragmented through ultrasonication, the fragments were purified and repaired. A base was added to the 3′end of the fragments and connected to an adapter required for the Illumina sequencing platform, thereby forming a complete sequencing template. Subsequently, the DNA fragment size was selected using methods such as agarose gel electrophoresis to remove unsuitable fragments and PCR amplification to increase the number of fragments in the library. The constructed sequencing library was subjected to quality control, and metagenomic sequencing was performed at the Illumina sequencing center.

### 2.13 Metabolomics analysis

The plasma metabolome was analyzed through gas chromatography-tandem mass spectrometry (GC-MS/MS; GC/MSD-7890B–5977B, Agilent Technologies, US) and liquid chromatography-tandem mass spectrometry (LC-MS/MS; UHPLC-Q-Exactive Orbitrap-MS, Agilent Technologies, US).

Blood was centrifuged for 10 min (4°C, 14,000 rpm). In total, 100 μL serum was mixed with 300 μL acetonitrile, vortexed for 1 min, and centrifuged for 10 min (4°C, 14,000 rpm). Then, 200 μL of the supernatant was collected. After drying the supernatant with nitrogen gas, 200 μL of acetonitrile was added to the dried sample for reconstitution, vortexed. The supernatant was used for injection analysis.

The raw data were processed using Compound Discoverer 3.3 software, and the metabolomics data were imported into SIMCA 14.1 software for multivariate data analysis. Differential metabolites were selected based on the basis of variable importance parameters (VIP >1) and t-tests (*p* < 0.05). Differential compounds were then identified and compared using the HMDB database (https://hmdb.ca/). Metabolic pathway analysis and screening were performed using MetaboAnalyst 5.0 (https://www.metaboanalyst.ca/).

0.2 g fecal sample was added to methanol to form a homogenate and centrifuged. The supernatant was collected, mixed with methanol, centrifuged again, and filtered. The final supernatant was collected and frozen.

Gas chromatography-mass spectrometry (GC/MS) analysis was conducted with an HP-5MS capillary column (30 m × 0.25 mm × 0.25 μm, Agilent J&W). 1-μL sample was automatically injected with a split ratio of 10:1, high-purity helium gas (<99.999%) being used as the carrier gas at a 1 mL/min flow rate and inlet temperature of 290°C. The temperature program: the column temperature: 40°C, 2 min; 300°C, 5 min. The mass spectrometry conditions included an EI ionization source and a scan range of m/z 40–400. Kovats retention indices were used to calculate compound retention times. The MassHunter quantification software was used for the automatic identification and integration of ion fragments. SCFA standards, including those for propionic acid (wkq20042911) and butyric acid (wkq20092401) were used for comparing retention times and corresponding MS spectra with the samples to confirm the SCFAs and calculate the concentrations.

### 2.14 Data processing

The raw data were processed using Compound Discoverer 3.3 to batch-extract compound peaks. After normalization, the data were imported into the SIMCA-P 13.0 statistical software package from Umetrics. The principal component analysis (PCA) and orthogonal partial least-squares discrimination analysis (OPLS-DA) were performed. Differential biomarkers were screened using t-tests. Differential metabolites were identified by combining them with the HMDB database (https://hmdb.ca/). GraphPad Prism 8.0.2 software was used to analyze the trend of changes in each metabolite. Metabolic pathways of the differential metabolites were analyzed using MetaboAnalyst 5.0 (https://www.metaboanalyst.ca/), and the involved metabolic pathways were visualized using KEGG (https://www.genome.jp/kegg/).

The Spearman rank correlation coefficient was employed to conduct the correlation analysis, to assess the strength of association between different taxonomic levels of microorganisms and resistance genes. We used |R| > 0.3 as the screening criterion, indicating that only correlations with an absolute correlation coefficient greater than 0.3 were considered to be of substantive significance. Furthermore, the *t*-test to compare the obtained correlation coefficients against this screening criterion to determine if there were significant differences. *p* < 0.05 was considered to be statistically significant in the correlation coefficient.

The data were presented as mean ± Standard error of the mean. One-way analysis of variance was used for multifactorial comparisons in this study. All data analysis was conducted with the SPSS 22.0 statistical software package (SPSS Inc, Chicago, IL, USA).

## 3 Results

### 3.1 Extraction and structural characterization of ESP

ESP was extracted from *E. sinica* Stapf, and the optimal extraction conditions for ESP were tested. Table 1 presents the monosaccharide composition of ESP.

**TABLE 1:** Composition of ESP.

No.	Name	Molecular formula	MW (g/mol)	Content (mg/kg)	Structure
1	D-Mannose	C_6_H_12_O_6_	180.16	2828.57	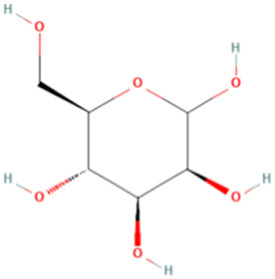
2	Glucosamine	C_6_H_13_NO_5_	179.17	460.00	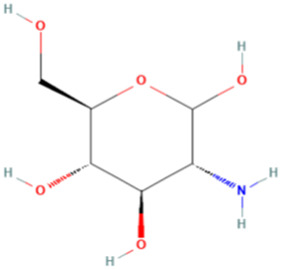
3	Ribose	C_5_H_10_O_5_	150.13	1,057.14	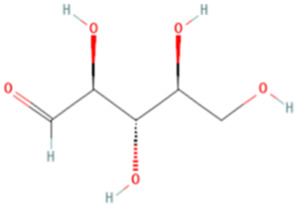
4	L-Rhamnose monohydrate	C_6_H_14_O_6_	182.17	2674.29	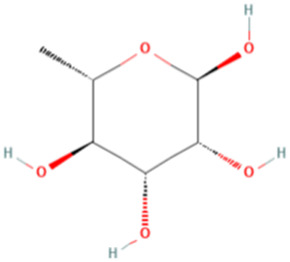
5	D-Glucuronic acid	C_6_H_10_O_7_	194.14	3,334.29	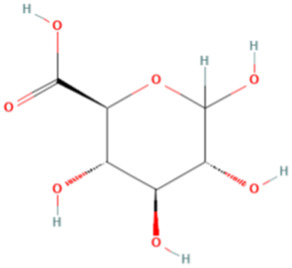
6	D-Galacturonic acid	C_6_H_10_O_7_	194.14	11,214.29	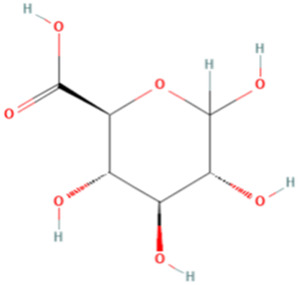
7	Glucose	C_6_H_12_O_6_	180.16	33,622.86	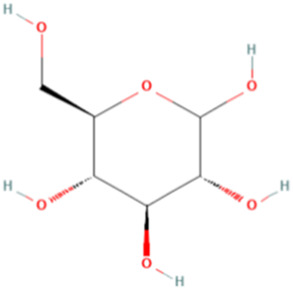
8	Galactose	C_6_H_12_O_6_	180.16	5500.00	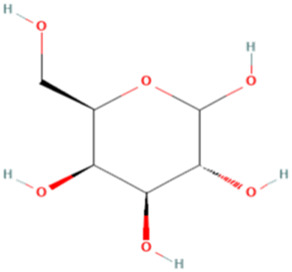
9	D-Xylose	C_5_H_10_O_5_	150.13	651.43	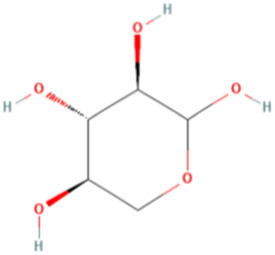
10	Arabinose	C_5_H_10_O_5_	150.13	10,140.00	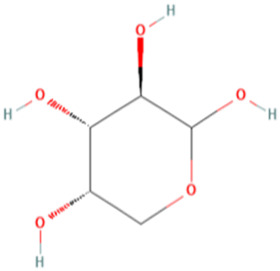
11	Fucose	C_6_H_12_O_5_	164.16	102.86	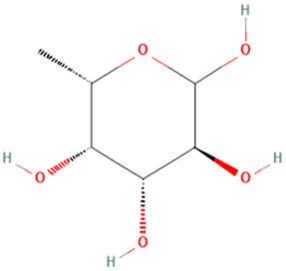

### 3.2 ESP significantly improves the general condition of mice

**FIGURE 1:**
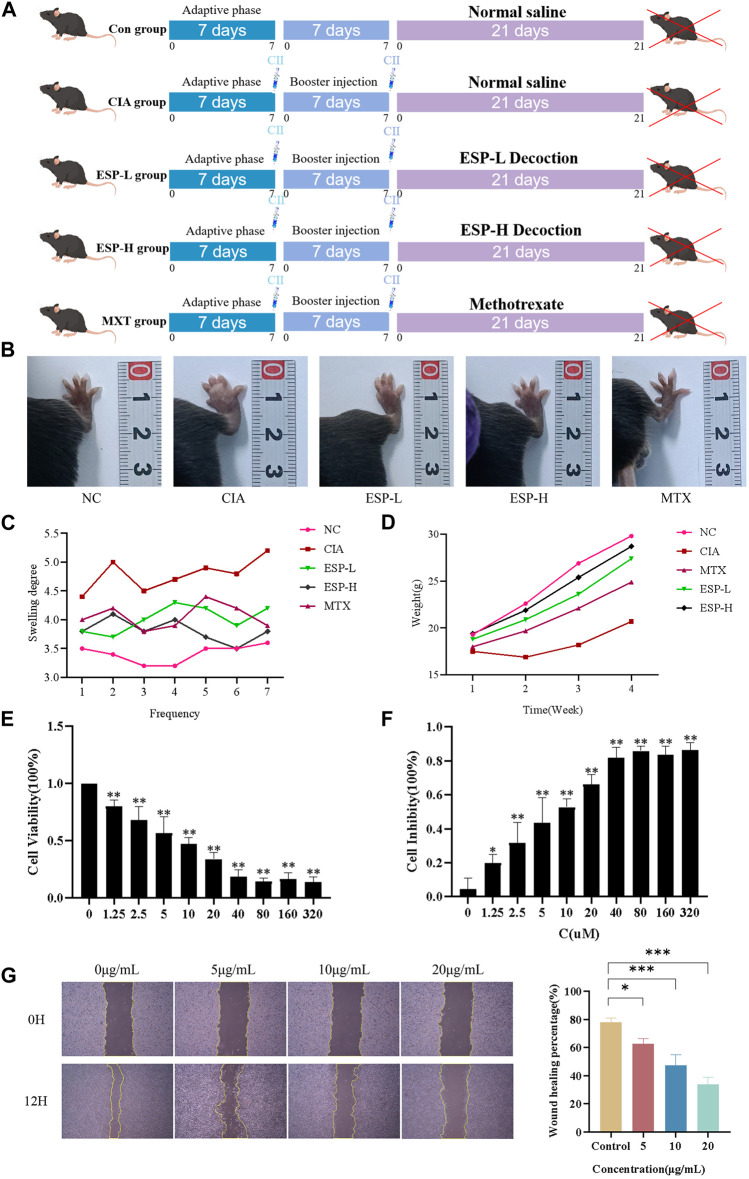
The role of ESP in the occurrence of rheumatoid arthritis. Timeline of animal experiments; **(B)** The effects of ESP on joint swelling in collagen-induced arthritis (CIA) rats; **(C–D)** Changes in the body weight and paw swelling in mice from each group; **(E–F)** Statistical analysis of MTT test results after treatment with different concentrations of ESP. **(G)** Statistical analysis of the impact of different concentraotins of ESP treatment on cell proliferatio(*p < 0.05; **p < 0.01 vs. 0 μM group).

To determine the preventive effect of ESP on RA occurrence, ESP was orally administered to the type II collagen-immunized C57BL/6J mice (Figure 1A). ESP dose-dependently inhibited the RA incidence, as evidenced by the significant increase in the thickness of the CIA left hind paw compared with the NC group (Figure 1B). Treatment of CIA mice with ESP (200, 400 mg/kg) and MTX gradually reduced the paw thickness (Figure 1C). After the mice were induced with collagen, their body weights exhibited a trend of stagnation or even decrease, and then gradually increased after 14 days. By contrast, the mice in the NC group displayed a continuous increase in body weight (Figure 1D). Importantly, no adverse reactions were observed in the ESP-treated mice, and high-dose ESP did not affect spleen and thymus weights (Supplementary Figure S1A). ESP-H treatment significantly suppressed cell proliferation (Figures 1E–G).

### 3.3 ESP prevents impairment of joint bone, cartilage, and intestinal barrier from RA

**FIGURE 2:**
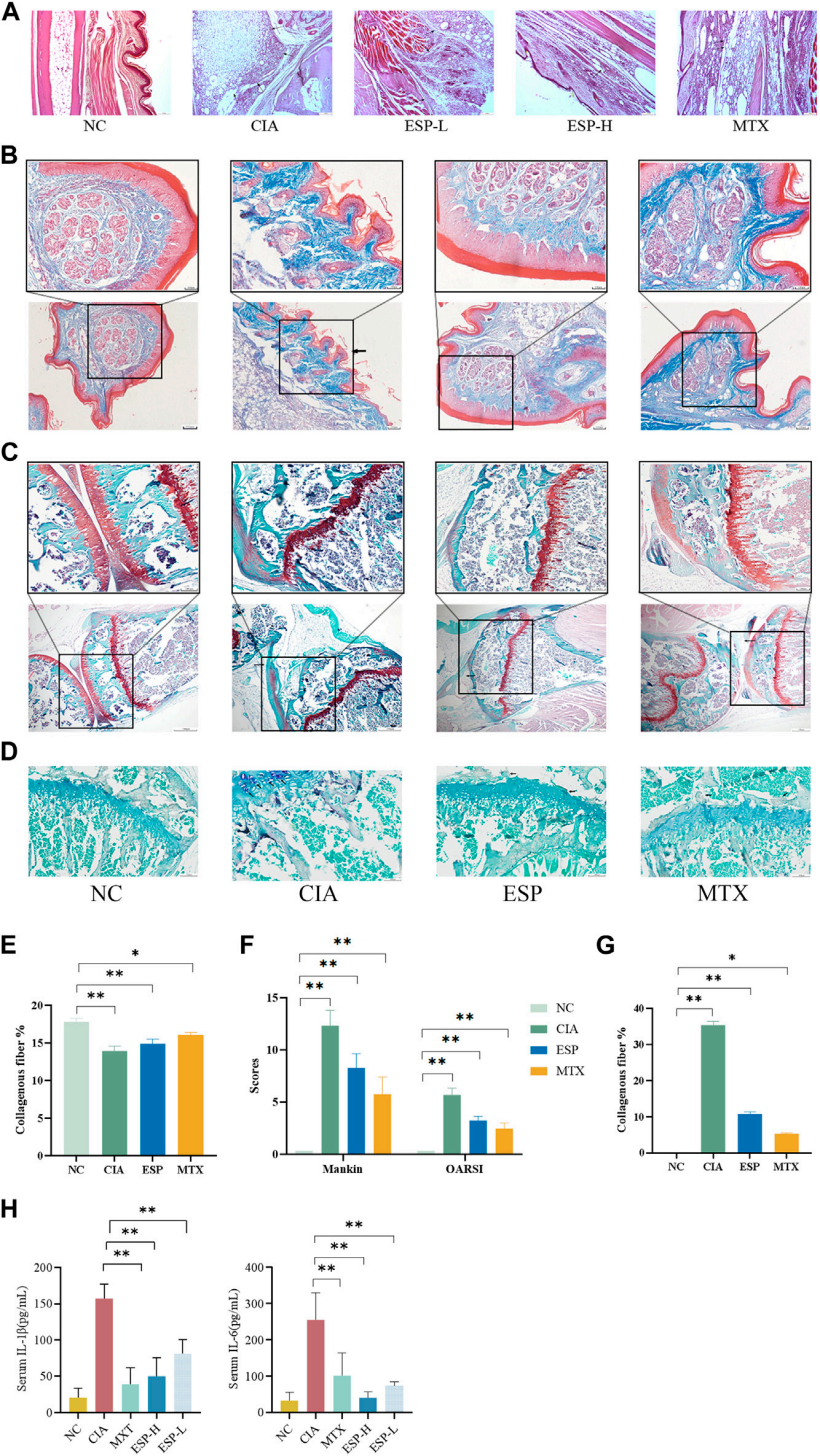
Effects of ESP on histopathology of joint tissues and immune organ indicators. **(A)** H&E staining of mice ankle joints; **(B.E)** Masson staining and statistical analysis; **(C,F)** Safranin O/Fast Green staining and Mankin cartilage tissue score, OARSI joint pathology score; **(D,G)** TRAP staining; **(H)** the IL-1β and IL-6 levels in the serum. (*p < 0.05; **p < 0.01)

The impact of type II collagen on joint bone and cartilage damage was evaluated through histopathological staining. H&E staining of mice ankle joints revealed significant infiltration of inflammatory cells, fibroblast proliferation, and neovascularization in the CIA mice, with uneven surfaces noted on the articular cartilage and local vascular shading (Figure 2A). Masson staining unveiled damage to the joint cartilage surface in the CIA group, with a disordered arrangement of collagen fibers and unclear boundaries between the cartilage and subchondral tissues (Figures 2B,E). Safranin O/Fast Green staining demonstrated inflammatory changes in the cartilage tissues of the CIA group, loss of the normal structure, surface defects, synovial tissue proliferation, and intermittent tide lines. The staining results were evaluated based on the basis of the Mankin cartilage tissue score and OARSI joint pathology score (Figures 2C,F). TRAP staining unveiled numerous positively stained osteoclasts in the joints of the CIA mice (Figures 2D,G). The RA-affected cartilage injury in the joints can accelerate synovial inflammation by producing pro-inflammatory cytokines (Koepsell, 2020). ESP treatment alleviated synovial inflammation in type II collagen-treated mice and reduced joint damage. ESP dose-dependently reduced the serum levels of the pro-inflammatory factors IL-1β and IL-6 in the CIA mice (Figure 2H), consistent with the *in vitro* experimental results. These findings indicate that ESP has a strong anti-inflammatory effect.

**FIGURE 3:**
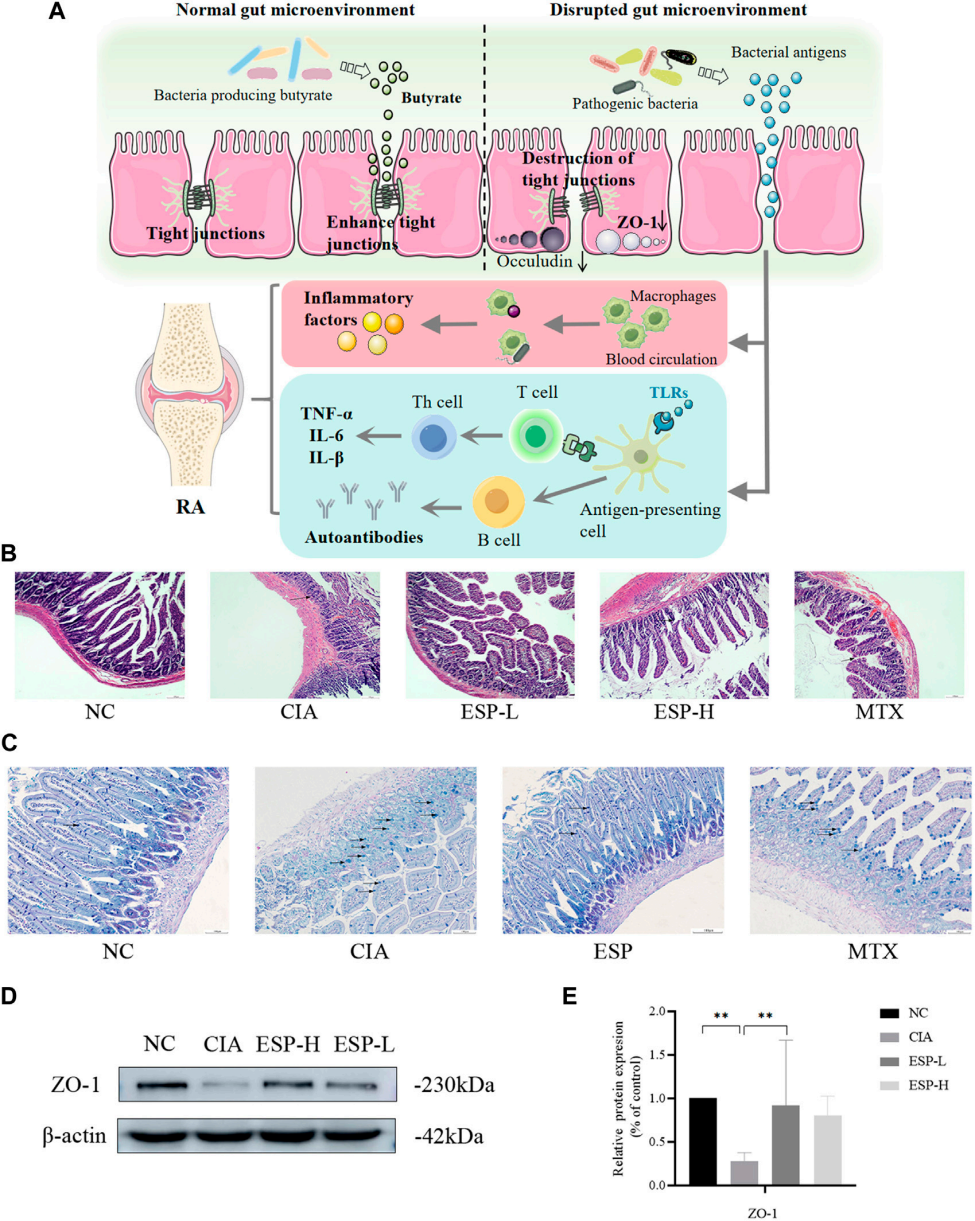
ESP restores intestinal barrier function in rheumatoid arthritis. **(A)** The link between the intestinal barrier and rheumatoid arthritis. After decreased secretion of ZO-1, the tight junctions are disrupted, leading to increased permeability. This allows the gut microbiota to enter the lamina propria through intestinal epithelial cells, leading to the production of inflammatory factors. Simultaneously, these microorganisms or their derived metabolites can enter the joints through the bloodstream; **(B)** H&E staining of mice intestinal tissues; **(C)** AB-PAS staining of mice intestinal tissues; **(D,E)** Immunoblotting detection of zonula occludens-1 (ZO-1) expression after ESP intervention. (*p < 0.05; **p < 0.01).

Gut dysbiosis might trigger the breakdown of gut barrier integrity and the leakage of microbiota-derived metabolites into gut tissue and even venous or lymphatic circulation, enabling exposure of the immune cells to bacterial antigens leading to local and systemic inflammation, increased pro-inflammatory cytokines such as IL-1 and IL-6 (Romero-Figueroa et al., 2023) (Figure 3A). The H&E staining (Figure 3B) and AB-PAS (Figure 3C) results revealed severe epithelial damage, inflammatory cell infiltration, and mucosal injury in the CIA mice. These histopathological abnormalities were largely restored in the ESP-treated mice. The expression of the tight junction protein ZO-1 significantly decreased in the colon of the CIA mice. However, this expression markedly increased after ESP treatment (Figures 3D,E).

### 3.4 ESP exerts protective effects on synovial cells in CIA mice through the TLR4/HDAC/NF-κB signaling pathway

**FIGURE 4:**
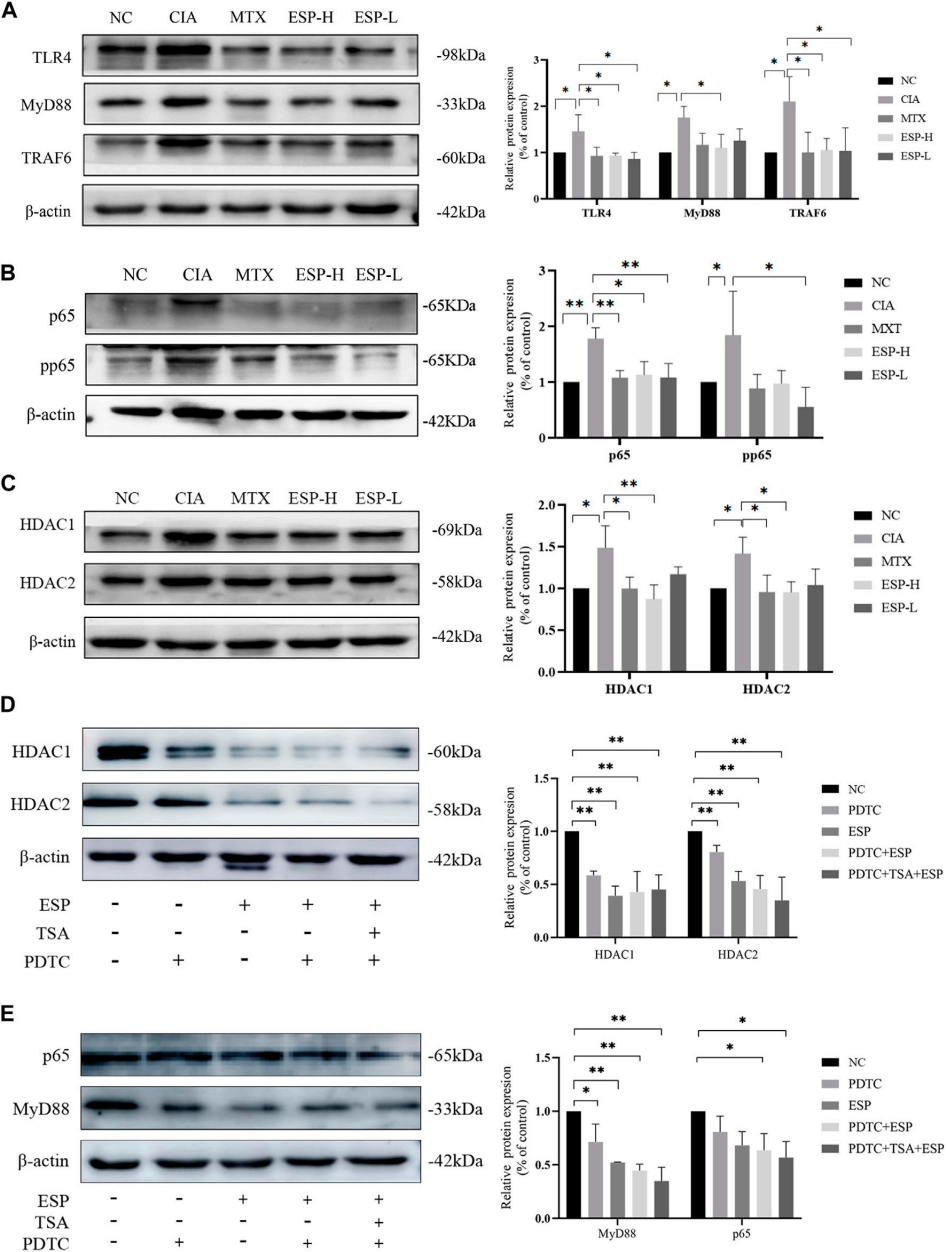
ESP exerts protective effects on CIA mice synovial cells through the TLR4/HDAC/NF-κB signaling pathway. **(A)** Immunoblotting detection of inflammatory proteins (TLR4, MyD88, TRAf6) expressions after ESP intervention; **(B,C)** Immunoblotting detection of the NF-κB signaling pathway proteins (p65, pp65, HDAC1, HDAC2) expressions after ESP intervention; **(D,E)** Immunoblotting detection of p65, MyD88, HDAC1, and HDAC2 protein expressions after ESP intervention and HDAC1/2 inhibitor TSA, NF-κB inhibitor PDTC intervention. (*p < 0.05; **p < 0.01).

The expression levels of inflammatory proteins in the mice synovial tissue were evaluated through western blotting. Compared with the CIA group, the ESP treatment group exhibited significantly reduced TLR4, MyD88, and TRAF6 expression (Figure 4A). ESP inhibited p65 and phosphorylated p65 expression in the NF-κB signaling pathway (Figure 4B), thus blocking HDAC1 and HDAC2 signals (Figure 4C). As shown in cell experiments, the HDAC1/2 inhibitor TSA (100 nM) and NF-κB inhibitor PDTC (100 nM). TSA and PDTC partially inhibited the HDAC inflammatory expression in the synovium (Figures 4D,E).

### 3.5 ESP reverses gut dysbiosis in RA

The metagenomic sequencing analysis revealed a pro-inflammatory shift in the colonic microbiota of the CIA model, with an increase in the abundance of gram-negative bacteria containing immunogenic LPS in their cell walls (Deng et al., 2021). The abundance of bacteria with pro-inflammatory characteristics (*Alistipes, Enterococcus, Enterorhabdus, Odoribacter*, and *Escherichia*) was higher, whereas the ESP intervention caused the diversity of the intestinal microbiota in the CIA model improved (Supplementary Figure S1B–F), and an increase in the abundance of anti-inflammatory bacteria (*Dubosiella, Roseburia, Bifidobacterium, Clostridium, Pseudoramibacter, Faecalibaculum,* and *Parabacteroides*).

**FIGURE 5:**
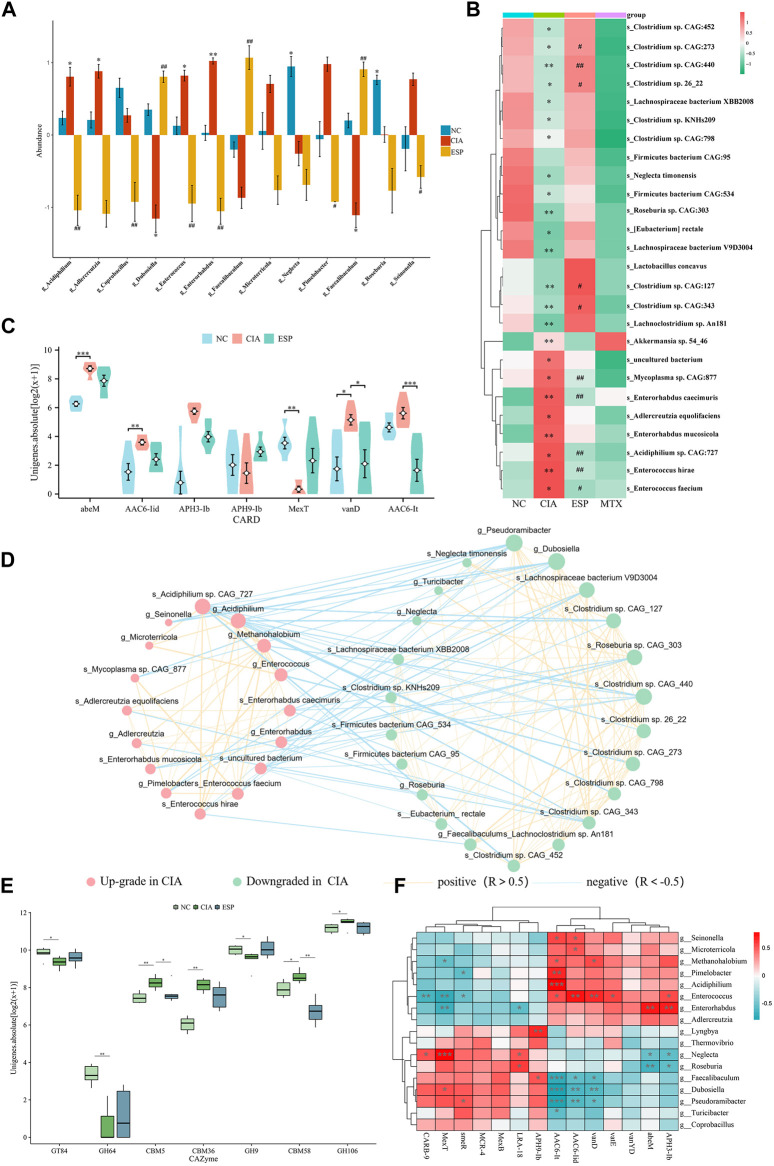
ESP regulates the gut microbiota in rheumatoid arthritis. **(A,B)** LEfSe analysis of changes in the gut microbiota abundance at the genus level **(A)** and species level; **(B)** between the groups of mice (*p < 0.05; **p < 0.01 vs NC group; ^*#*^ *p* < 0.05; ^##^ *p* < 0.01 vs CIA group); **(C)** statistical analysis of differences in resistance genes between the groups of mice; **(D)** ecological network of interactions between differentially abundant bacteria at the genus and species levels; **(E)** comparison of carbohydrate-active enzyme abundance in the mice between the groups; **(F)** Heatmap depicting the correlation between differentially abundant bacteria at the genus level and differentially resistant genes (*p < 0.05; **p < 0.01).

The LEfSe analysis identified a higher abundance of *g_Enterococcus, g_Enterorhabdus, g_Alistipes, g_Odoribacter, s_Enterorhabdus caecimuris, s_Escherichia coli, *and* s_Enterococcus faecium* in the CIA group. After the ESP treatment, the abundance of *g_Dubosiella, g_Pseudoramibacter, g_Bifidobacterium, g_Faecalibaculum, s_Dubosiella newyorkensis, s_Clostridium* sp. *26_*22, *s_Bifidobacterium pseudolongum,* and *s_Parabacteroides chinchillae* significantly increased in the gut microbiota of mice compared with the CIA group, consistent with the abundance in the NC group (Figures 5A,B; Supplementary Figure S1G,H).

Furthermore, an interaction ecological network constructed from the inter-group differential microbiota, thereby revealing the existence of mutual exclusion relationships between harmful and beneficial bacteria in the gut microbiota of the CIA mice (Figure 5D). Metagenomic sequencing revealed a relative increase in carbohydrate-active enzymes (CAZymes) CBM5, CBM36, and CBM58 in the CIA group (Figure 5E).

By analyzing the microbial correlations of antibiotic resistance genes (ARGs) (Figures 5C,F), we found a negative correlation between APH3-lb, abeM, and *g_Roseburia*, and a positive correlation with *g_Enterorhabdus*. AAC6-lt was negatively correlated with *g_Dubosiella* and *g_Pseudoramibacter* and positively correlated with *g_Enterococcus*. These datas suggested that ESP counteracts pro-inflammatory bacteria such as *g_Enterococcus* and *s_Enterococcus hirae*, thereby improving RA-induced microbiota dysbiosis.

### 3.6 ESP modulates gut metabolites in RA

Given the major role of gut microbiota metabolites in regulating health and disease, a metabolomic analysis was conducted using the fecal samples from the mice (Supplementary Figure S2).

**FIGURE 6:**
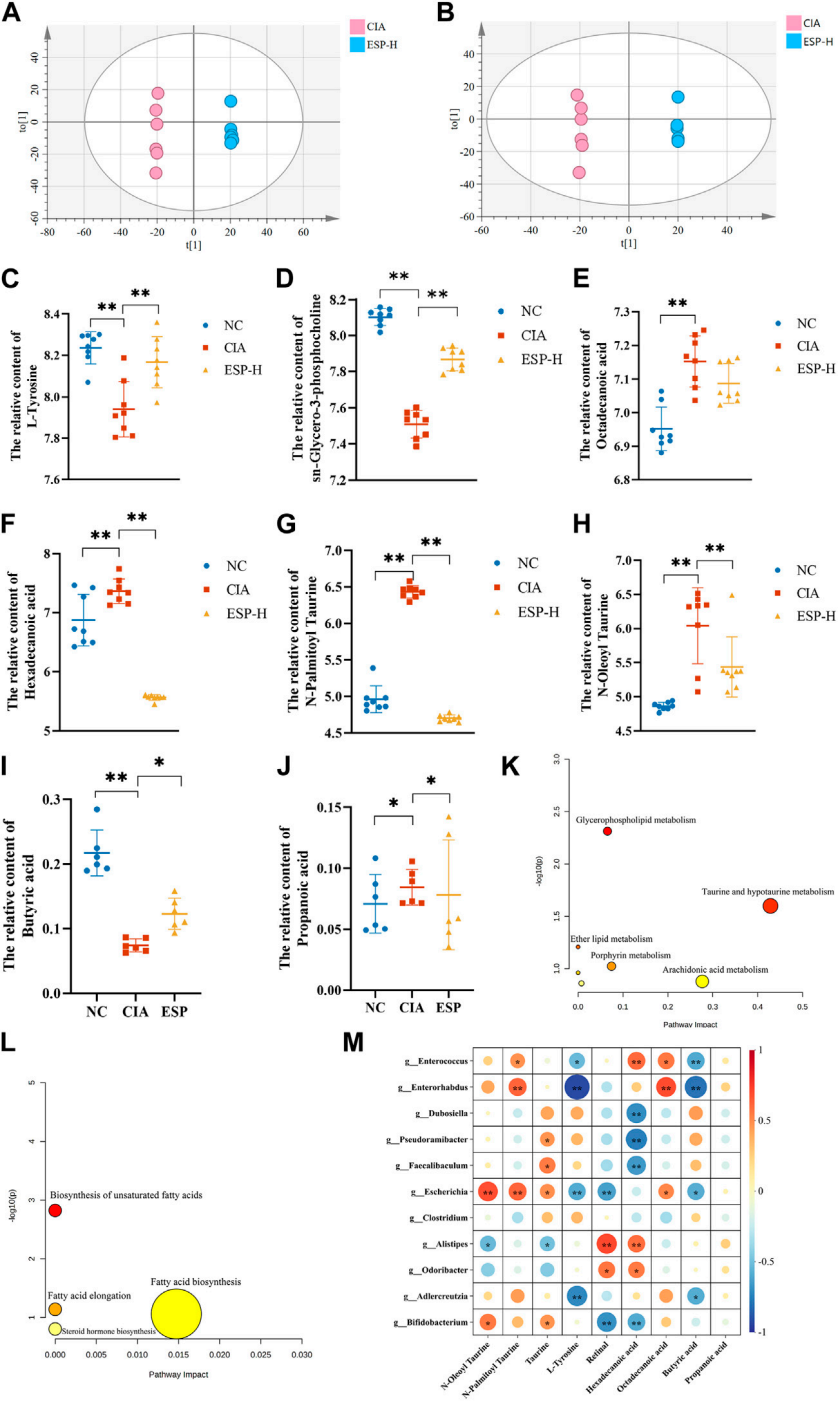
ESP regulates intestinal metabolites in rheumatoid arthritis. **(A,B)** Orthogonal partial least squares discriminant analysis (OPLS-DA) of the CIA group and ESP-H group under the positive ion mode **(A)** and negative ion mode **(B)**; **(C–H)** Changes in the content of different metabolites between the groups; **(I,J)** Changes in the content of different short-chain fatty acids between the study groups; **(K,L)** Pathway analysis of differential metabolites; **(M)** Heatmap depicting the correlation between differentially abundant bacteria at the genus level and differential metabolites (*p < 0.05; **p < 0.01).

In the positive ion mode (Figure 6A), compared with the NC group, the levels of differential metabolites such as L-tyrosine and sn-glycero-3-phosphocholine decreased in the CIA group. In the negative ion mode (Figure 6B), the content of metabolites such as hexadecanoic acid, octadecanoic acid, N-oleoyl taurine, and N-palmitoyl taurine were reduced, and these trends were reversed after ESP administration (Figures 6C–H). The concentration of butyric acid in the fecal of mice induced by type II collagen decreased, while the concentration of propionic acid increased. Treatment with ESP was found to elevate the levels of butyric acid and reduce the levels of propionic acid (Figures 6I,J; Supplementary Figure S3).

The selected differential metabolites were inputted into the MetaboAnalyst 5.0 database for the pathway analysis. The pathways associated with the serum differential metabolites, including taurine and hypotaurine metabolism, porphyrin metabolism, arochidonic acid metabolism, and fatty acid biosynsis, exhibited a high correlation, which indicated their potential relevance to ESP therapy for RA. Figure 6K–L presents the metabolic pathway diagram (Detailed metabolites listed in Supplementary Material S2). It is found that butyric acid and L-Tyrosine are negatively correlated with *Enterococcus* and *Enterorhabdus*. Hexadecanoic acid is negatively correlated with *Dubosiella*, *Pseudoramibacter,* and *Bifidobacterium*, while positively correlated with *Enterococcus* (Figure 6M). It is speculated that the bacterial community may play a role through the action of secondary metabolites on the TLR4/HDAC/NF-κB signaling pathway.

## 4 Discussion

An interaction occurs among local inflammation, imbalanced gut microbiota, and host immune dysregulation in RA pathogenesis. (Eicher and Mohajeri, 2022). Microbiota-derived metabolites participate in signal transduction, maintenance of the mucosal barrier, and immune system regulation, thereby serving as key factors in the host-microbiota crosstalk in a pathogenic environment. Thus, they become a new participant in the mucosal immune function and inflammation, playing a crucial role in RA occurrence and development (Wolter et al., 2021). Traditional Chinese medicine comprises various monosaccharides, polysaccharides, and other carbohydrates, and so, a large amount of enzymes is required to facilitate their biological conversion. However, humans have a limited number of genes encoding CAZymes. The gut microbiota can encode a diverse and extensive CAZymes library. It thus breaks down and metabolizes a large amount of complex polysaccharides into SCFAs, regulates host immunity, and produces antioxidative substances.

Plant cell walls are composed of polysaccharides, and glycoside hydrolases (GHs) typically catalyze plant cell wall deconstruction (Hong et al., 2023). Medicinal plant fibers are often large-molecule polysaccharides promoting bacterial growth adept at fiber degradation (Ye et al., 2022).

Our monosaccharide mass spectrometry results revealed that ESP contains high levels of glucuronic acid, galactose, arabinose, and glucose. The immunomodulatory activity of polysaccharides depends on their molecular weight and monosaccharide composition. For example, arabinose and glucose contents in polysaccharides are related to significantly enhanced immunoreactivity (Wastyk et al., 2021). ESP alleviated synovial inflammation in the CIA mice and had a protective effect on type II collagen-induced joint damage, which manifested as significant improvements in body weight, paw swelling, and histological scores, and reduced IL-1β and IL-6 levels.

Polysaccharides exert a significant regulatory effect on the ecological imbalance of the gut microbiota. ESP greatly improved the CIA-induced gut microbiota imbalance in the mice. Activation of the NF-κB signaling pathway is associated with the *in vitro* immunomodulatory effects of polysaccharides (Wang et al., 2023a; Wang et al., 2023b). TLR4 expression activates the NF-κB pathway, thereby stimulating the release of inflammatory enzymes (Chang et al., 2024).

The mucosal barrier maintains the normal dynamic balance of the intestinal environment. This structure primarily includes the mucosal barrier, intestinal epithelial barrier, and immune barrier (Yang et al., 2023). The mucus layer protects intestinal epithelial cells and serves as a crucial medium for host-bacteria interactions. The colonic mucus barrier serves as the primary line of defense against intestinal pathogens. The reduced thickness and chemical composition of the mucus layer increases the ability of bacteria to penetrate the mucous barrier (Sang et al., 2023). ZO-1 is crucial for mucosal healing and epithelial barrier function, and its loss can induce defects in the repair process (Kuo et al., 2021). In the present study, ESP promoted intestinal barrier repair in the CIA mice, improved the permeability of the ileal and colonic mucosa, and inhibited intestinal inflammation.

In our study, the elevation of serum IL-1β and IL-6 levels in the CIA mice was restored after ESP treatment. This indicated that ESP could inhibit the entry of inflammatory factors from the intestine into the joints. Additionally, increased TLR4 expression in the CIA mice synovial inflammation activated the NF-κB pathway. This promotes the release of inflammatory enzymes and leads to joint and cartilage destruction, along with IL-1β- and IL-6-induced excessive osteoclast production. Furthermore, the analysis revealed that intestinal inflammation-induced elevation in HDAC1 and HDAC2 levels led to NF-κB phosphorylation, and TLR4 and MyD88 activation in the RA synovial tissue, which are critical factors in RA immune imbalance. Ultimately, ESP alleviates RA by suppressing inflammation in the intestine and synovium.

In the CIA model, the abundance of bacteria such as *Alistipes*, *Enterococcus*, and *Enterorhabdus* was higher, which led to or exacerbated infections. *Enterococcus* is a major cause of multidrug-resistant infections (Xiong et al., 2022). It can reshape the metabolic environment and serve as a source of fermented amino acids like *C. difficile*, which then promotes the persistence of infections (Smith et al., 2022). After ESP treatment was administered, the abundance of *Dubosiella, Bifidobacterium, Clostridium, Pseudoramibacter,* and other bacteria increased. *Clostridium* is a bacterium with strong butyrate-producing ability. It enhances the integrity of the epithelial barrier and inhibits inflammation. *Dubosiella newyorkensis* plays a crucial role in enhancing mucosal barrier integrity and regulating Treg/Th17 balance. It also alleviates host mucosal and extraintestinal inflammation by producing SCFAs (especially propionate) (Zhang et al., 2024). Evidence from ESP intervention-involving experiments confirms an increase in butyrate. *Bifidobacterium* can reduce the incidence and severity of RA (Jiang et al., 2023). *B. pseudolongum* regulates enhanced immune therapeutic responses by producing the metabolite guanosine. *B. pseudolongum* induces Th1 differentiation, triggers IFN-γ production by CD4^+^ and CD8^+^ T cells (Mager et al., 2020), decomposes indigestible carbohydrates, and improves intestinal barrier integrity. Intestinal inflammation is associated with impaired lipid absorption, and an increase in *Bifidobacterium* abundance may help alleviate inflammation and increase lipid absorption (Weninger et al., 2023).

The carbohydrate-binding module (CBM) is a type of multi-module enzyme protein that responds to polysaccharide reactions and has a high affinity for tyrosine (Liu et al., 2022). CBMs typically attach to CAZymes to enhance their catalytic activity and are expressed on the surface of pathogenic proteins (Liberato et al., 2022). CAZymes include GHs, polysaccharide lyases, carbohydrate esterases, and lytic polysaccharide monooxygenases (Attia and Brumer, 2021). Infectious and inflammatory diseases are partially attributable to abnormal signal transduction involving various protease actions. The gut is rich in various proteases sourced from the host, microbes, and diet. Proteolytic activity must be strictly regulated to prevent tissue damag e (Crits-Christoph et al., 2022; Edwinson et al., 2022).

The gut microbiota also serves as a reservoir for ARGs (Crits-Christoph et al., 2022). Bacteria frequently exchange ARGs within the human microbiome, with the gut bacterial community being a hub for horizontal gene transfer (Carvalho et al., 2022). Chromosomally encoded efflux pumps are the major determinants of antibiotic resistance in *Pseudomonas aeruginosa*. MexT is a transcriptional activator of one such efflux pump in *P. aeruginosa*, which leads to increased antibiotic resistance (Kim et al., 2019). Bacteria can encode aminoglycoside-modifying enzymes such as acetyl-CoA-dependent aminoglycoside acetyltransferase and ATP/GTP-dependent phosphotransferase (APH). APH confers high levels of resistance to aminoglycoside antibiotics through enzymatic modification (Smith et al., 2019). The APH gene expression gradually increases with an increase in the arabitol concentration, and this increase is consistent with resistance phenotypes (Zhang et al., 2016). Aminoglycoside phosphotransferases can phosphorylate specific hydroxyl groups in aminoglycoside antibiotics (e.g., tobramycin and kanamycin), which are highly clinically relevant antibiotics, thereby rendering them very effective inactivators (Adam et al., 2024). AbeM was upregulated in imipenem-resistant *Acinetobacter baumannii* isolates (Choquet et al., 2021).

Metabolomics study showed that several metabolites displayed significant differences in enrichment between the CIA and ESP groups, including taurine, N-oleoyl taurine, and N-palmitoyl taurine. Taurine levels in the ESP group were significantly reduced. Taurine levels in the blood of patients with RA-related autoimmune diseases, such as systemic lupus erythematosus (Li et al., 2020) and primary Sjögren’s syndrome (Herrala et al., 2020), increase, and their levels are positively correlated with disease activity. N-Oleoyl taurine increased and N-palmitoyl taurine levels decreased in the ESP group. Serum N-oleoyl taurine promotes proliferation, abnormal differentiation, and impaired cell apoptosis, with taurine being its core structure (Huang et al., 2019). N-palmitoyl taurine can induce cell migration and upregulate inflammatory metabolic pathways (Reinke et al., 2017). In our study, ESP upregulated butyrate levels in the CIA group. Butyrate is the most widely studied HDAC inhibitor that can promote Tconvs, inhibit Tregs and osteoclasts, suppress HDAC expression, and downregulate pro-inflammatory cytokine levels (He et al., 2022).

Our results demonstrated that ESP administered to the II collagen-induced mice significantly increased SCFA levels, whereas restored the anti-inflammatory cytokine levels and intestinal barrier function. Therefore, we speculated microbial metabolites regulate the balance and immune homeostasis of RA.

HDACs are crucial for various inflammatory diseases, with HDAC-1 capable of downregulating inflammatory signals to inhibit the expression of NF-κB-regulated genes. HDAC-2 does not directly participate in NF-κB signal transduction, but it regulates HDAC-1-associated NF-κB activity. HDAC-1 inhibition in intestinal epithelial cells induces increased phosphorylation and nuclear localization of p65 NF-κB. HDAC inhibitors maintain a balance between pro-inflammatory and anti-inflammatory gene expression (Pooladanda et al., 2019; He et al., 2022).

In conclusion, our study demonstrates that ESP can effectively reduce IL-1β and IL-6 levels, suppress inflammation in RA, inhibit tumor-like proliferation of synovial cells, counteract NF-κB phosphorylation, and protect against joint damage in the RA model. An integrated analysis of metagenomics and LC-MS/MS and GC-MS/MS metabolomics indicated that the potential mechanisms of ESP treatment in CIA are enriched in taurine and hypotaurine metabolism, porphyrin metabolism, and the enrichment of SCFA-producing bacteria such as *Dubosiella, Bifidobacterium, Clostridium*, and *Pseudoramibacter*. Furthermore, metabolites involved in metabolic pathways may contribute to the protective effects of ESP on CIA.

## Data Availability

The datasets presented in this study can be found in online repositories. The names of the repository/repositories and accession number(s) can be found in the article/Supplementary Material.
