# Supplementary material for: Ephedra sinica polysaccharide regulate the anti-inflammatory immunity of intestinal microecology and bacterial metabolites in rheumatoid arthritis
Source: Front Pharmacol. 2024 May 23;15:1414675. doi: 10.3389/fphar.2024.1414675 (PMC11153800; doi:10.3389/fphar.2024.1414675)
Supplement: Supplementary file 1 [file DataSheet3.docx]

**Chromatographic column** (ACQUITY BEH C18100 mm x 2.1 mm, 1.7 um)

**Mass spectrometry-grade methanol** (CAS: 67-56-1, Thermo Fisher Scientific (China) Co., Ltd.)

**Mass spectrometry-grade acetonitrile** (CAS: 75-05-8, Thermo Fisher Scientific (China) Co., Ltd.)

**Bovine type II collagen** (Product number: 220195, SIGMA-ALDRICH (SHANGHAI) Trading Co., Ltd.)

**Freund's Complete Adjuvant** (Product number: SLCL9648, Sigma-Aldrich)

***Tripterygium wilfordii* tablets** (Product number: Z42021212, Huangshi Feiyun Pharmaceutical Co., Ltd.)

**Toll-like receptor 4** (Product number: A5258, ABclonal)

**p65 subunit of NF-κB** (Product number: A2547, ABclonal)

Myeloid differentiation primary response 88 (Product number: 67969-1-Ig, Proteintech)

**Mouse Interleukin-1β (IL-1β) ELISA Kit** (Product number: JL18442, Shanghai Future Industry Co., Ltd.)

**Mouse Interleukin-6** (IL-6) ELISA Kit (Product number: JL20268, Shanghai Future Industrial Co., Ltd.)

**Methotrexate tablets** (Product number: 036210103, Shanghai Sine Pharmaceutical Laboratories Co., Ltd.)

**PDTC** (Product number: 5108-96-3, MedChemExpress)

**TSA** (Product number: 58880-19-6, MedChemExpress)
