# Supplementary material for: Ephedra sinica polysaccharide regulate the anti-inflammatory immunity of intestinal microecology and bacterial metabolites in rheumatoid arthritis
Source: Front Pharmacol. 2024 May 23;15:1414675. doi: 10.3389/fphar.2024.1414675 (PMC11153800; doi:10.3389/fphar.2024.1414675)
Supplement: Supplementary file 2 [file DataSheet2.docx]

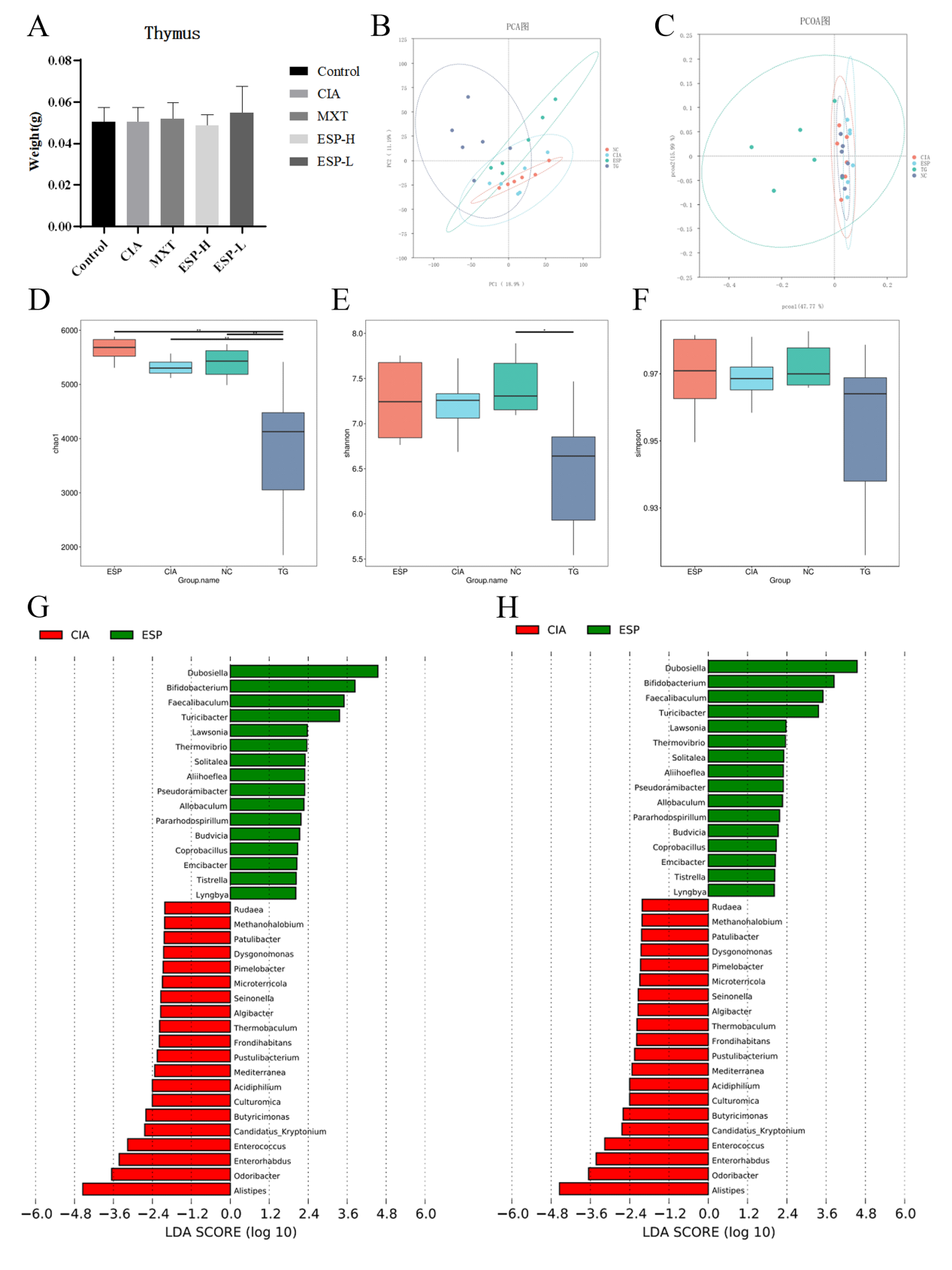


Supplementary Figure 1 (A). Changes in the thymus weight of the mice after intervention in each group; (B, C) Alpha diversity analysis of metagenomic sequencing, (B) PCA (Principal Component Analysis), (C) PCoA (Principal Coordinates Analysis); (D–F) Beta diversity analysis of metagenomic sequencing, (D) Chao1 index, (E) Shannon index, (F) Simpson index; (G, H) LEfSe analysis of metagenomic sequencing, (G) At the genus level, (H) At the species level.


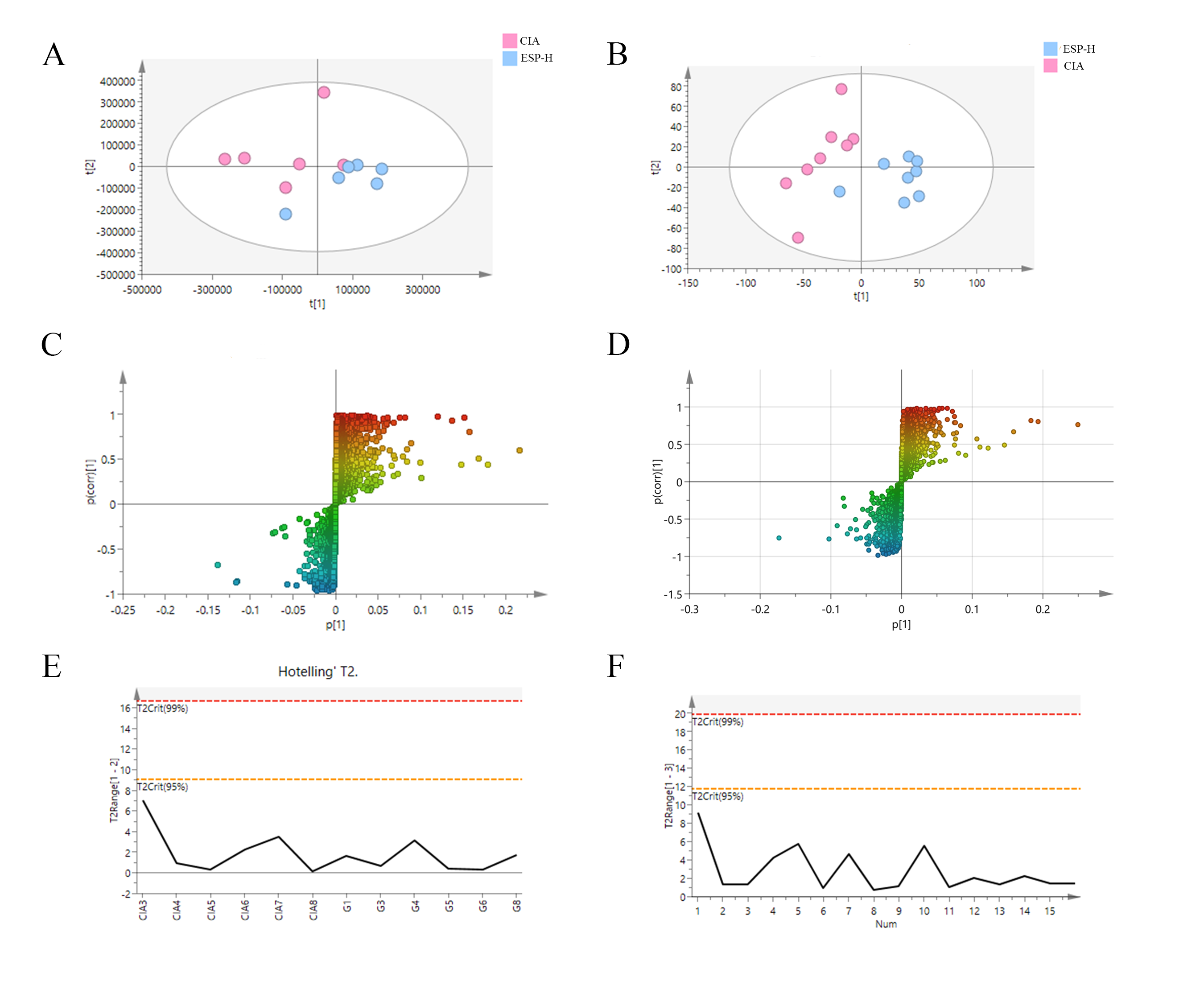


Supplementary Figure 2 (A, B) Principal component analysis of the CIA group and ESP-H group under positive and negative ion modes; (C, D) S-plot of the CIA group and ESP-H group under positive and negative ion modes; (E, F) Hotelling' T2 outlier detection under positive and negative ion modes.


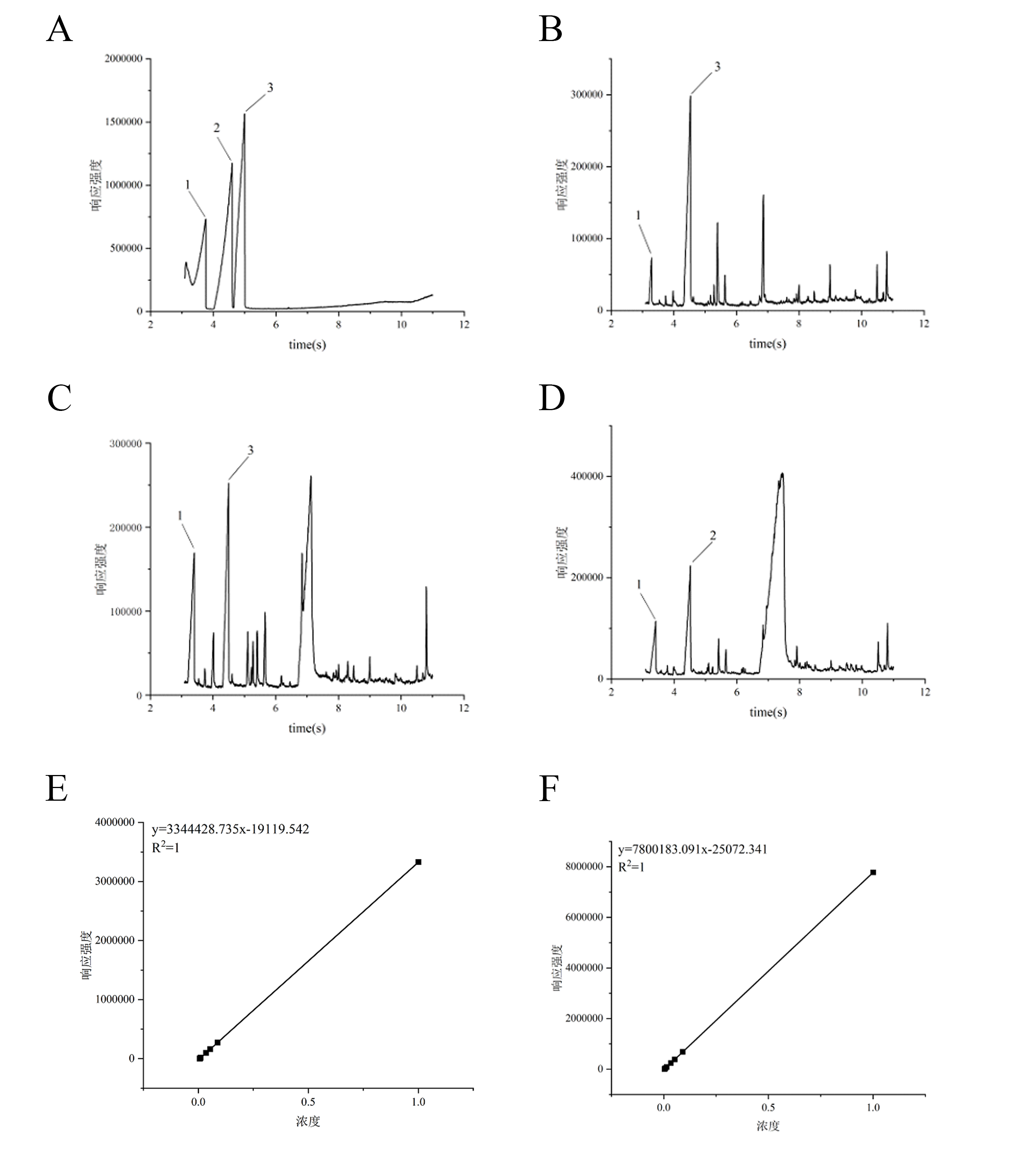


Supplementary Figure 3 (A–D) Qualitative analysis of gas chromatography-mass spectrometry data, (A) Standard sample group, (B) CIA group, (C) ESP group, (D) MTX group (1. Propionic acid, 2. Isobutyric acid, 3. Butyric acid); (E, F) Quantitative standard curves of data; (E) Propionic acid, (F) Butyric acid.
