# Supplementary material for: Ephedra sinica polysaccharide regulate the anti-inflammatory immunity of intestinal microecology and bacterial metabolites in rheumatoid arthritis
Source: Front Pharmacol. 2024 May 23;15:1414675. doi: 10.3389/fphar.2024.1414675 (PMC11153800; doi:10.3389/fphar.2024.1414675)
Supplement: Supplementary file 3 [file DataSheet1.docx]

**1. Pre-processing Method**

(1) Weigh an appropriate amount of the sample into a hydrolysis tube, add 1 mL of 2 mol/L trifluoroacetic acid, purge the content with nitrogen, and hydrolyze at 110°C for 5 h in an oven. Remove the tube, cool it to room temperature, and transfer 0.5 mL of the content into a 4-mL centrifuge tube, followed by adjusting the pH to neutral.

(2) Add 0.2 mL of 0.3 mol/L NaOH and 0.4 mL of PMP methanol solution to the 4 mL centrifuge tube, purge the content with nitrogen, and incubate the tube in a 70°C water bath for 60 min. After cooling the tube to room temperature, add 0.2 mL of 0.3 mol/L HCl to it, make the content volume 2 mL with water, add 1.5 mL of chloroform, shake the content well, and allow the tube to stand for stratification. Now, discard the lower chloroform layer, and repeat this extraction procedure three times, followed by filtering the aqueous layer through a 0.45-μm filter membrane and analyzing.

**2. Instrument Method**

(1) The data acquisition instrument system includes an Agilent 1200 equipped with a UV detector.

(2) Chromatographic column: C18 4.6 mm × 250 mm × 5 μm.

(3) Mobile phase A: 15% acetonitrile (acetonitrile diluted with 0.05 mol/L KH_2_PO_4_ to pH 6.8), Mobile phase B: 40% acetonitrile (acetonitrile diluted with 0.05 mol/L KH_2_PO_4_ to pH 6.8).

(4) Gradient of the mobile phases:

| Time (min) | Flow rate(mL/min) | A% 15% acetonitrile | B% 40% acetonitrile |
| --- | --- | --- | --- |
| 0.0 | 1.0 | 100 | 0 |
| 10.0 | 1.0 | 92 | 8 |
| 40.0 | 1.0 | 63 | 37 |
| 45.0 | 1.0 | 100 | 0 |
| 50.0 | 1.0 | 100 | 0 |

(5) Flow rate: 1.0 mL/min； Column temperature: 25℃； Injection volume: 20 μL； Detection wavelength: 254 nm

**3. Calculation formula**

Monosaccharide content was calculated according to the following formula:

W—Content of the target analyte in the sample, unit mg/kg;

C—Concentration of the target analyte in the test sample, unit mg/L;

C_0_—Concentration of the target analyte in the blank control, unit mg/L;

V—Final volume, unit mL;

N—Dilution factor;

m—Sample sampling amount, unit g.

**References:**

1. Jayamanohar, Jabastin, et al. "Prebiotic potential of water extractable polysaccharide from red kidney bean (Phaseolus vulgaris L.)." *Lwt* 101 (2019): 703-710.
2. Lee, Shin Ja, et al. "Effects of Gelidium amansii extracts on in vitro ruminal fermentation characteristics, methanogenesis, and microbial populations." *Asian-Australasian journal of animal sciences* 31.1 (2018): 71.
3. Yang, Tsung-Han, et al. "The anti-obesity effect of polysaccharide-rich red algae (Gelidium amansii) hot-water extracts in high-fat diet-induced obese hamsters." *Marine drugs* 17.9 (2019): 532.
